# The impact of economic growth on health care utilization: a longitudinal study in rural Vietnam

**DOI:** 10.1186/1475-9276-12-19

**Published:** 2013-03-16

**Authors:** Nguyen Thi Minh Thoa, Nguyen Xuan Thanh, Nguyen Thi Kim Chuc, Lars Lindholm

**Affiliations:** 1 Hanoi Medical University, 01-Ton That Tung Str, Dong Da Dist, Hanoi, Vietnam; 2 Institute of Health Economics, Edmonton, Alberta, Canada; 3 Umeå International School of Public Health, Umeå, Sweden

**Keywords:** Economic growth, Health care utilization, Household expenditure Vietnam

## Abstract

**Introduction:**

In many developing countries, including Vietnam, out-of-pocket payment is the principal source of health financing. The economic growth is widening the gap between rich and poor people in many aspects, including health care utilization. While inequities in health between high- and low-income groups have been well investigated, this study aims to investigate how the health care utilization changes when the economic condition is changing at a household level.

**Method:**

We analysed a panel data of 11,260 households in a rural district of Vietnam. Of the sample, 74.4% having an income increase between 2003 and 2007 were defined as households with economic growth. We used a double-differences propensity score matching technique to compare the changes in health care expenditure as percentage of total expenditure and health care utilization from 2003 to 2005, from 2003 to 2007, and from 2005 to 2007, between households with and without economic growth.

**Results:**

Households with economic growth spent less percentage of their expenditure for health care, but used more provincial/central hospitals (higher quality health care services) than households without economic growth. The differences were statistically significant.

**Conclusions:**

The results suggest that households with economic growth are better off also in terms of health services utilization. Efforts for reducing inequalities in health should therefore consider the inequality in income growth over time.

## Introduction

Health is considered as a fundamental human right and the achievement of the highest possible level of health is one of the most important worldwide social goals [1]. This can be partly attributed to the fact that poor health can have a significant economic impact on any households. Poor health can make households property exhausted, indebted, and reduce their essential consumption [2] because people with poor health are not only having productivity and income losses, but also out-of-pocket (OOP) expenses for needed healthcare services.

The implementation of user fees is likely a barrier to access adequate health services in poor settings. Some opinions suggest that user charges can generate vital resources at the local level and helps to provide better quality services [3]; however, others opinions highlight its’ negative effects, particularly the inequity for the poor people [4].

In many low- and middle-income countries, the level of government spending on health is low compared with other sectors and OOP expenditure is the principal source of health financing in those nations. OOP expenditure accounts for more than 80% of the private expenditure on health in many developing countries [5] which likely has catastrophic economic effects on individuals and their families, as well as limits their possibilities to receive adequate healthcare [2] In Vietnam, total health expenditure (THE) in 2008 was 7.3% of Gross Domestic Product (GDP), with government expenditure accounting for only 38.5% of total health budget [5,6]. The introduction of “Doi Moi”, the new economic reform that transforms Vietnam from a centrally planned economy to a socialist-oriented market economy in 1986, increased the OOP health expenditures as a proportion of private health expenditures from 59% in 1989 to 84% in 1998 and to 90.2% in 2007 [2,7]. Vietnamese households have not been able to hold their food and non-food consumption constant due to income reductions and extra medical care expenditure [8,9]. Hence, healthcare expenses have become a financial burden and influenced healthcare service seeking behavior, especially among the poor.

In Vietnam, Ministry of Health is responsible for the professional management of both public and private health sectors through health departments in each province/city/district. There are four different levels of healthcare services, including central, provincial, district, and commune level, where central is the highest and commune is the lowest level of care. People are encouraged to firstly use services at the commune level for both preventive and curative care and they will be referred to a higher level of care if needed.

The private healthcare services in Vietnam have developed rapidly since 1993 when the law on private pharmaceutical and medical practice was launched. Since 1993, when there were no private health services, this sector has grown to about 83 private hospitals, 30,000 private clinics and 9,000 private pharmacies in 2008. With a growing number, the private sector plays an increasingly important role in the Vietnam health system [10].

Since the “Doi Moi”, the economy has grown rapidly and is being integrated into the world’s economy [11]. According to World Health Organization Statistics, the GDP per capita of Vietnam increased from $610 in 1990 to $2,700 (PPP international $) in 2008 and Vietnam has become one of the most potent markets in the Southeast Asia [7,11]. Living standards have also been changed as the result of economic growth and the gap between rich and poor people has been increased as the consequence of the market economy. The poor is a high-risk group of people in many aspects in the society, including health and healthcare. Several studies indicate that the gap between the rich and the poor is large in healthcare spending and utilization, and that the higher socioeconomic status is correlated with better health and longer life [12-14]; however, little is known about how people use healthcare services when their economic condition changes year by year.

This study aims to investigate the association between healthcare utilization and economic growth among households in Ba Vi district in Vietnam from 2003 to 2007 and to try to answer the question “Is there any change in healthcare utilization when the income is changing?”

## Materials and methodology

### Materials

Ba Vi is a rural district located in the North of Vietnam with a population of approximately 240,000 and covering an area of 410km2 with various geographies, including lowland, midland and mountainous areas. Children under one year of age comprise 1.5% of the overall population, children under 5 years of age 7.9%, and women aged 15 to 49 years 27.1%. .Agricultural production and livestock breeding are the main economic activities of the local people (81% of population). The mMajor products are wet rice, cassava, corn, soya beans, green beans and fruits such as pineapple, mandarin orange and papaya. Other economic activities are forestry (8%), fishing (1%), small trade (3%), handicraft (6%) and transport (1%) [12,15].

There is only one district healthcare center, 3 policlinics, and 32 commune health stations. All communes have implemented the primary healthcare programs, including expanded immunization and acute respiratory infections, family planning, and antenatal care. In addition, there were approximately 90 licensed private health facilities (private clinics and pharmacies) in Ba Vi in 2009 [16-18].

In 1998, the “Epidemiological Field Laboratory for Health Systems Research” in Ba Vi district, called “FilaBavi”, was established with the overall objective to develop an epidemiological surveillance system which could provide basic health and healthcare data, supply evidence for health policymakers, serve as a background and sampling frame for specific studies, and constitute a setting for epidemiological training of research students [15].

Sample size of FilaBavi was calculated based on an estimated infant mortality rate (IMR) of 45 per 1,000 live births aiming to detect a change in IMR of 15 per 1,000 after three years of study. A random sampling of clusters (each cluster is generally a village), with probability proportional to population size in each unit, was performed, and 67 clusters with a reported population size of 51,024 inhabitants in 11,089 households were selected from the total 352 clusters in the district [15,18]. The total number of households has changed each year due to migration.

The baseline survey started in January 1999, from which the re-census surveys were performed every second year, gathering information on socio-economic characteristics of households, including housing conditions, water resources, latrines, healthcare expenditure, total expenditure, total income, agricultural land, access to the nearest commune health station and hospital, and household socio-economic status (SES) as classified by the local leaders. The follow-up surveys were performed quarterly gathering information on demographic (e.g. age, sex, ethnicity, religion, occupation, education, marital status), morbidity (e.g. number of sick episodes) and healthcare utilization (e.g. number of use of different types of healthcare providers) of household members in the last three months. A detail of the FilaBavi longitudinal demographic surveillance site has been published elsewhere [15].

In this study, we used the data of 3 re-censuses and 12 quarterly follow-up surveys in 2003, 2005 and 2007. In total, this study followed 11,260 households continuously from 2003 to 2007.

## Methods

We analyzed data at a household level using a double-difference propensity matching technique [19-21]. Based on the change of income between 2003 and 2007 we grouped the sample into two groups: households with and without economic growth (EG). Households without EG were “treated” in terms of propensity score matching (a more general term would be “exposed”). As controls, we tried to find “untreated” (not exposed) households as similar as possible to the treated ones. This was done by matching the propensity scores. The propensity score measures the similarity between “treated” and “untreated” in terms of a vector of observable characteristics. This score is the probability of a household not having EG. The two groups should be as similar as possible in pre-treatment characteristics, implying that differences in outcomes can be attributed to the treatment. To estimate the propensity scores we used a logistic regression. “Treated” and “untreated” cases were matched according to their propensity scores. We selected the nearest (in terms of the propensity score) “untreated” neighbors to a “treated” case. Finally, we compared the difference in change during the studied periods between “treated” and “untreated” households for the chosen outcome variables. We used a balance test to make sure the bias was reduced after matching.

The chosen outcome variables were the HCE as a percentage of total expenditure; the numbers of use of public healthcare facilities, including communal health station (CHS), district hospital (DH) and provincial/central hospitals (these were analyzed separately); the number of use of private healthcare services, including private practitioners of western and/or traditional medicine; the number of self-treatment, and the number of go-to-pharmacies to buy drugs with or without advices of the pharmacists or drug sellers.

Variables for estimating the propensity scores to match the treated with the control households were household-head characteristics, including sex, age, religion, ethnicity, marital status, education, and occupation; and household characteristics, including total household expenditure, distance to CHS (km), to DH (km), households economic status, number of members, number of males, number of children under 6 years old, number of people 60 years or older, number of sick episodes, number of sick person, number of restricted days cause by sickness, etc. These variables were selected based on potential association between them and the probability of being treated, but also on their availability in the dataset.

Stata software version 9.2 (StataCorp, College Station, Texas, USA) was used for analyses.

### Ethical consideration

This study was carried out within the Health System Research Project collaborated between Vietnam and Sweden. Permission was obtained from the Ministry of Health of Vietnam, and local authorities. Additionally, informed consent was obtained from inhabitants as well. Ethical approval for the overall surveillance activities, including data collection on vital statistics, was approved by the Research Ethics Committee at Umea University.

## Results

In total, there were 11,260 households that have information in all the 3 years for analysis. Of these, 2,884 households did not have income growth between 2003 and 2007.

Table 1 shows a description of the outcome variables by the EG/non-EG and years. Households with EG used slightly more services than households without EG in both public and private health facilities in 2005 and 2007; however, their expenditure for health accounted for a smaller percentage of total household expenditure. Self-treatment was the most common health care option accounting for about 60% of the total number of healthcare utilization, followed go-to-pharmacies (19%), by private (15%), DH (7%), CHC (6%), and P/CH (3%).

**Table 1:** Description of outcome variables by the EG/non-EG groups and years

	**EG**		**Non EG**	
	**Mean**	**SD**	**Mean**	**SD**
**2003**				
HCE as % of total expenditure (%)	5.486	10.873	5.360	10.494
Number of self-treatment (#)	6.593	4.983	6.712	4.962
Number of go-to-pharmacy (#)	1.181	1.844	1.103	1.775
Number of private health care utilizations (#)	1.959	2.295	1.992	2.269
Number of CHS utilizations (#)	0.453	1.150	0.414	1.088
Number of DH utilizations (#)	0.326	0.785	0.304	0.715
Number of P/CH utilizations (#)	0.132	0.450	0.130	0.436
Total number of utilizations (#)	10.644	7.661	10.655	7.422
**2005**				
HCE as % of total expenditure (%)	4.938	10.301	5.558	12.056
Number of self-treatment (#)	5.607	4.128	5.575	4.208
Number of go-to-pharmacy (#)	1.150	1.922	1.045	1.862
Number of private health care utilizations (#)	1.774	2.217	1.757	2.121
Number of CHS utilizations (#)	0.460	1.080	0.395	0.976
Number of DH utilizations (#)	0.342	0.825	0.326	0.741
Number of P/CH utilizations (#)	0.121	0.424	0.130	0.451
Total number of utilizations (#)	9.450	6.635	9.227	6.609
**2007**				
HCE as % of total expenditure (%)	4.888	10.450	5.455	11.454
Number of self-treatment (#)	5.806	4.080	5.606	3.993
Number of go-to-pharmacy (#)	1.356	1.967	1.345	2.005
Number of private health care utilizations (#)	1.789	2.215	1.675	2.148
Number of CHS utilizations (#)	0.475	1.107	0.399	1.011
Number of DH utilizations (#)	0.587	1.146	0.584	1.187
Number of P/CH utilizations (#)	0.155	0.477	0.144	0.463
Total number of utilizations (#)	10.168	6.943	9.754	6.869

*EG* Economic Group, *HCE* Health Care Expenditure, *CHS* Commune Health Station, *DH* district hospital, *P/CH* Province/Center Hospital.

Logistic regression estimating the propensity scores for matching is shown in Table 2. The LR Chi^2^ test showed that the model variables jointly explained which households were more likely to be treated as non-EG households [p (LR Chi^2^ (21) = 223.10) = 0.000]. The probability of a household being in non-economic growth group depended significantly at the 5% level on age, education and religion of the heads of households, and on the number of restricted days caused by sickness, the number of sick episodes, the distances from households to the DH, and the total expenditures of household.

**Table 2:** Logistic regression to estimate propensity scores

**Dependent variable: non-EG households**				
**Independent variables**	**Coef.**	**[95% Conf.**	**Interval]**	**Sig.**
Total household expenditure	−0.0017	−0.0026	−0.0086	Yes
Distance to commune health center	0.0111	−0.0246	0.0468	No
Distance to district hospital	0.0502	0.0365	0.0639	Yes
Household economic status	−0.0471	−0.1000	0.0056	No
Number of household member	−0.0218	−0.0708	0.0272	No
Number of males in household	0.0389	−0.0353	0.1132	No
Number of children under 6 years in household	0.0603	−0.0555	0.1762	No
Number of elder people over 60 years in household	0.0579	−0.0761	0.1920	No
Number of sick episodes	0.0294	0.0046	0.0543	Yes
Number of sick person	−0.0406	−0.1149	0.0338	No
Number of restricted days caused by sickness	−0.0029	−0.0051	−0.0006	Yes
Number of children under 6 years who were sick	−0.0153	−0.1447	0.1141	No
Number of elder people > 60 years who were sick	−0.0564	−0.2014	0.0886	No
Number of males who were sick	−0.0205	−0.1102	0.0691	No
Age of household head in years	−0.0095	−0.0138	−0.0052	Yes
Sex of household head	−0.0656	−0.2210	0.0899	No
Education of household head	0.0373	0.0230	0.0516	Yes
Religion of household head	−0.4345	−0.6806	−0.1883	Yes
Ethnic of households head	0.1283	−0.0900	0.3467	No
Married status of household head	−0.0427	−0.1009	0.0149	No
Occupation of household head	−0.0063	−0.0155	0.0029	No
Constant	1.6697	1.1617	2.1777	Yes

N=11,260; LR Chi^2^ (21) = 223.10; Prob> Chi^2^ =0.0000.

Table 3 shows the differences between households with and without EG in terms of the changes in healthcare utilization from 2003 to 2005, from 2005 to 2007, and from 2003 to 2007. The differences in changes of the total number of healthcare utilization between households with and without EG were not significant in all the three periods. Some significant differences were found in the utilization of health services, though.

**Table 3:** The relationship between economic growth and health care utilization

	**Changes between years**		**Difference in the changes**			
	**EG**	**Non EG**	**Mean**	**95% CI**		**Sig.**
**2003-2005**						
HCE as % of total expenditure (%)	−0.528	0.458	−0.987	−1.651	−0.398	Yes
Number of self-treatment (#)	−0.991	−1.094	0.103	−0.157	0.359	No
Number of go-to-pharmacy (#)	−0.030	−0.046	0.016	−0.099	0.101	No
Number of private health care utilizations (#)	−0.188	−0.204	0.016	−0.119	0.149	No
Number of CHS utilizations (#)	0.005	−0.046	0.052	−0.025	0.102	No
Number of DH utilizations (#)	0.016	0.019	−0.003	−0.046	0.033	No
Number of P/CH utilizations (#)	−0.010	0.007	−0.017	−0.042	0.012	No
Total number of utilizations (#)	−1.199	−1.365	0.167	−0.249	0.545	No
**2005-2007**						
HCE as % of total expenditure (%)	−0.065	0.222	−0.287	−1.066	0.560	No
Number of self-treatment (#)	0.196	−0.062	0.258	−0.103	0.484	No
Number of go-to-pharmacy (#)	0.209	0.182	0.027	−0.083	0.123	No
Number of private health care utilizations (#)	0.014	0.021	−0.007	−0.164	0.066	No
Number of CHS utilizations (#)	0.016	0.030	−0.013	−0.111	0.053	No
Number of DH utilizations (#)	0.247	0.269	−0.022	−0.093	0.047	No
Number of P/CH utilizations (#)	0.034	−0.005	0.039	0.016	0.075	Yes
Total number of utilizations (#)	0.716	0.434	0.282	−0.350	0.690	No
**2003-2007**						
HCE as % of total expenditure (%)	−0.588	0.640	−1.227	−2.312	−0.724	Yes
Number of self-treatment (#)	−0.797	−1.165	0.368	0.067	0.663	Yes
Number of go-to-pharmacy (#)	0.176	0.143	0.033	−0.070	0.124	No
Number of private health care utilizations (#)	−0.176	−0.190	0.014	−0.154	0.115	No
Number of CHS utilizations (#)	0.023	−0.015	0.038	−0.030	0.123	No
Number of DH utilizations (#)	0.262	0.280	−0.018	−0.088	0.043	No
Number of P/CH utilizations (#)	0.023	0.002	0.021	0.001	0.051	Yes
Total number of utilizations (#)	−0.488	−0.944	0.456	−0.013	0.825	No

*EG* Economic Growth, *CI* Confident Interval, *Sig.* Significant, *HCE* Health Care Utilization, *CHS* Commune Health station, *DH* District Hospital, *P/CH* Province/Center Hospital.

In the first period (2003–2005), only the difference in the change of HCE as percentage of total household expenditure was statistically significant. Households without EG spent significantly more percentage of their expenditure, for healthcare, than households with EG.

In the period between 2005 and 2007, the significance was found in the change of utilization of the higher level of healthcare. Households with EG used healthcare services at provincial/central hospitals significantly more than households without EG.

In the long-term, from 2003 to 2007, significant results were found in both HCE as percentage of total expenditure and the utilization of the higher level of healthcare. Specifically, households with EG spent significantly less percentage of their expenditure, for healthcare, but used significantly more provincial/central hospitals than households without EG.

## Discussion

This study provides a different viewpoint of the close link between economic status and healthcare utilization of households in rural Vietnam. Unlike others studies demonstrating a cross-sectional association between economic status and healthcare utilization [2,3,22], our results demonstrate a longitudinal relationship showing how healthcare utilization changes when the economic status is changing. Households with EG are better off in comparison with those without EG, in terms of both the healthcare expenditure as a percentage of total expenditure and the utilization of higher levels of healthcare (provincial or central hospitals) where the quality of care is higher. This result is consistent with other studies indicated that the better income groups typically utilize a higher level of public health services [13,23].

In a country where the inflation is high and the out-of-pocket payment is a main source of healthcare financing, such as is the case in Vietnam, it is not surprising that households without an increase of income over the years (for various reasons) have been worse off spending a larger share of the total household expenditure for healthcare, and reducing the use of higher quality, but more expensive healthcare services at the provincial/central hospitals. The poor health and the smaller share of expenditure for economic investment may in turn negatively impact their further earnings.

The most disadvantage group of people is the poor (according to the classification of local government) who have non-EG over time. This group accounted for (13.94%) our sample. They face a double barrier accessing health services; one is of course the poverty and another is non-EG. It is shown that poor people have more health problems but less assess to health services even for those who are provided a health insurance for the poor card. This is because of indirect costs, including traveling, accommodation, food and so on, since they usually live in remote areas [22]. This suggests health insurance only may not be sufficient help such individuals access health services.

Solutions should therefore be not only health insurance to reduce the out-of-pocket payment for healthcare, but also some kind of insurance for minimizing income reductions such as employment insurance, farming insurance, livestock insurance, and etc. The main occupation in Ba Vi district is farming and 53.8% of households head in the non-EG group are famers indicating farming might be a risky business in Ba Vi and therefore some kind of insurance for this could be valuable.

We used a similar analytical approach as the one used in Thanh et al. [23], but with different groups of the treated and controls (poor vs. non-poor in that study and EG vs. non-EG in our study). The double-difference propensity score matching technique could take care of time-invariant unobservable variables (fixed effects). The extent to which bias is reduced by the matching depends on the richness and quality of the control variables (i.e. the independent variables in the logistic regression). We used 21 control variables, of which 7 were significant.

One limitation to this study could be that as the data was collected by interviews, there may be some recall bias; however, we analyzed the overtime changes in the outcomes and there is no evidence to expect the bias impacts differently over the years.

## Conclusion

This study shows households with EG are better off in comparison with households without EG, in terms of both the healthcare expenditure as a percentage of total expenditure and the utilization of higher quality healthcare services. Efforts for reducing inequalities in health should therefore consider the inequality in income growth over time.

## Abbreviations

CHS: Commune health station; CI: Confident interval; DH: District hospital; EG: Economic growth; HCE: Healthcare expenditure; HCU: Healthcare utilization; HH: Households; OOP: Out-of-pocket payment; P/CH: Province/center hospital; SD: Standard deviation; SES: Social economic status; Sig: Significant; THE: Total health expenditure.

## Competing interests

The authors declare that they have no competing interests.

## Authors’ contributions

NTMT and NXT planned the study and performed statistical analyses. NTMT prepared the first draft of the manuscript. NTKC was responsible for data collection and management. LL supervised the whole process. NXT, NTKC, and LL provided comments and suggestions to NTMT to revise the manuscript. All the authors read and approved the final manuscript.
